# Cognitive frailty predicting all-cause mortality among community-living older adults in Taiwan: A 4-year nationwide population-based cohort study

**DOI:** 10.1371/journal.pone.0200447

**Published:** 2018-07-12

**Authors:** Wei-Ju Lee, Li-Ning Peng, Chih-Kuang Liang, Ching-Hui Loh, Liang-Kung Chen

**Affiliations:** 1 Aging and Health Research Center, National Yang Ming University, Taipei, Taiwan; 2 Department of Geriatrics, National Yang Ming University School of Medicine, Taipei, Taiwan; 3 Department of Family Medicine, Taipei Veterans General Hospital Yuanshan Branch, Yi-Land, Taiwan; 4 Center for Geriatrics and Gerontology, Taipei Veterans General Hospital, Taipei, Taiwan; 5 Center for Geriatrics and Gerontology, Kaohsiung Veterans General Hospital, Kaohsiung, Taiwan; 6 Center of Health and Aging, Hualien Tzu Chi Hospital Buddhist Tzu Chi Medical Foundation, Hualien, Taiwan; Ehime University Graduate School of Medicine, JAPAN

## Abstract

**Background:**

Cognitive frailty (CF) featured as frailty plus cognitive impairment was deemed to be a novel target for dementia and disable prevention. The study was intended to investigate the epidemiology of CF and the association between CF and all-cause mortality.

**Methods:**

The national representative cohort study was comprised of 1,103 community-living middle-aged and older adults. CF was defined as the co-existence of dynapenia (weakness and/or slowness) and cognitive impairment (1.5 standard deviations below the age-, sex- and education-matched norms in cognitive tests) without known neurodegenerative diseases. Dynapenia was defined by the Asian Working Group for Sarcopenia and cognitive function was assessed by the Short Portable Mental Status Questionnaire.

**Results:**

The prevalence of CF was 8.6% in this study. Subjects with CF were older, more likely to be women, having less regular exercise, fewer educational years, more depressive symptoms and greater multimorbidity. Compared to robust individuals, CF was significantly associated with all-cause mortality (HR: 3.1, 95% CI:1.3–7.7, p = 0.012).

**Conclusion:**

Dynapenia and cognitive impairment synergistically contribute to the mortality risk for the participants in this study. Further study is needed to explore the underlying pathophysiology and the reversibility of CF.

## Introduction

The concept of cognitive frailty (CF) was firstly proposed by Panza, et al., in 2006 to capture a complex phenotype of people by the concomitant presence of physical frailty and cognitive impairment for disability and dementia prevention [1]. The consensus from International Academy on Nutrition and Aging (I.A.N.A) and the International Association of Gerontology and Geriatrics (I.A.G.G) proposed the operational definition of CF as the co-existence of physical frailty (defined by Fried’s criteria) and mild cognitive impairment (Clinical Dementia Rating (CDR) scale = 0.5), and without dementia, and other neurodegenerative diseases [2]. The operational definition was proposed conceptually without supporting epidemiological evidences. After the introduction of the IANA/IAGG definition of CF, two major controversies were reported. First, the prevalence of CF defined by IANA/IAGG criteria was low, ranged from 1.2% to 1.8, which could not identify meaningful numbers for intervention [3]. Second, many MCI patients (CDR = 0.5) eventually presented with a progressive and irreversible process to dementia [4], and extensive or irreversible neural damages may have occurred already [1]. Hence, MCI may not be an appropriate component in the diagnostic criteria for CF due to the lack of benefits for dementia prevention [5].

A consensus from the Subjective Cognitive Decline Initiative Working Group proposed research criteria for cognitive performance testing for pre-MCI subjective cognitive decline, which was defined as lower or equal to 1.5 standard deviation from age- gender- and education-adjusted norms on standardized cognitive tests [6,7]. On the other hand, pre-frailty may achieve better outcomes when timely intervention was introduced and has become the main target for primary prevention despite that physical frailty *per se* has been shown to be a reversible state [8]. Some researchers argued that clinical manifestations of frailty phenotypes were initiated by weakness and slowness [9], and proposed pre-frailty to be the diagnostic component of CF [10]. Previous studies have identified that dynapenia, i.e. slowness and/or weakness, was an important subtype in frailty development and was substantially associated with adverse clinical outcomes [11–13]. Dynapenia, either in pre-frail or frail state, was also significantly associated with cognitive impairment [12]. Moreover, results from clustering analysis of brain MRI images showed that slowness and weakness were both associated with reduced gray matter in the cerebellum [13]. Altogether, selecting dynapenia plus cognitive impairment (1.5 SD below normal age- sex- and education-adjusted norms on standardized cognitive tests) to constitute the diagnostic criteria of CF may be a better approach. Therefore, this study aimed to explore the combined effects of dynapenia and cognitive impairment on all-cause mortality.

## Methods

### Study population and study design

**Fig 1:**
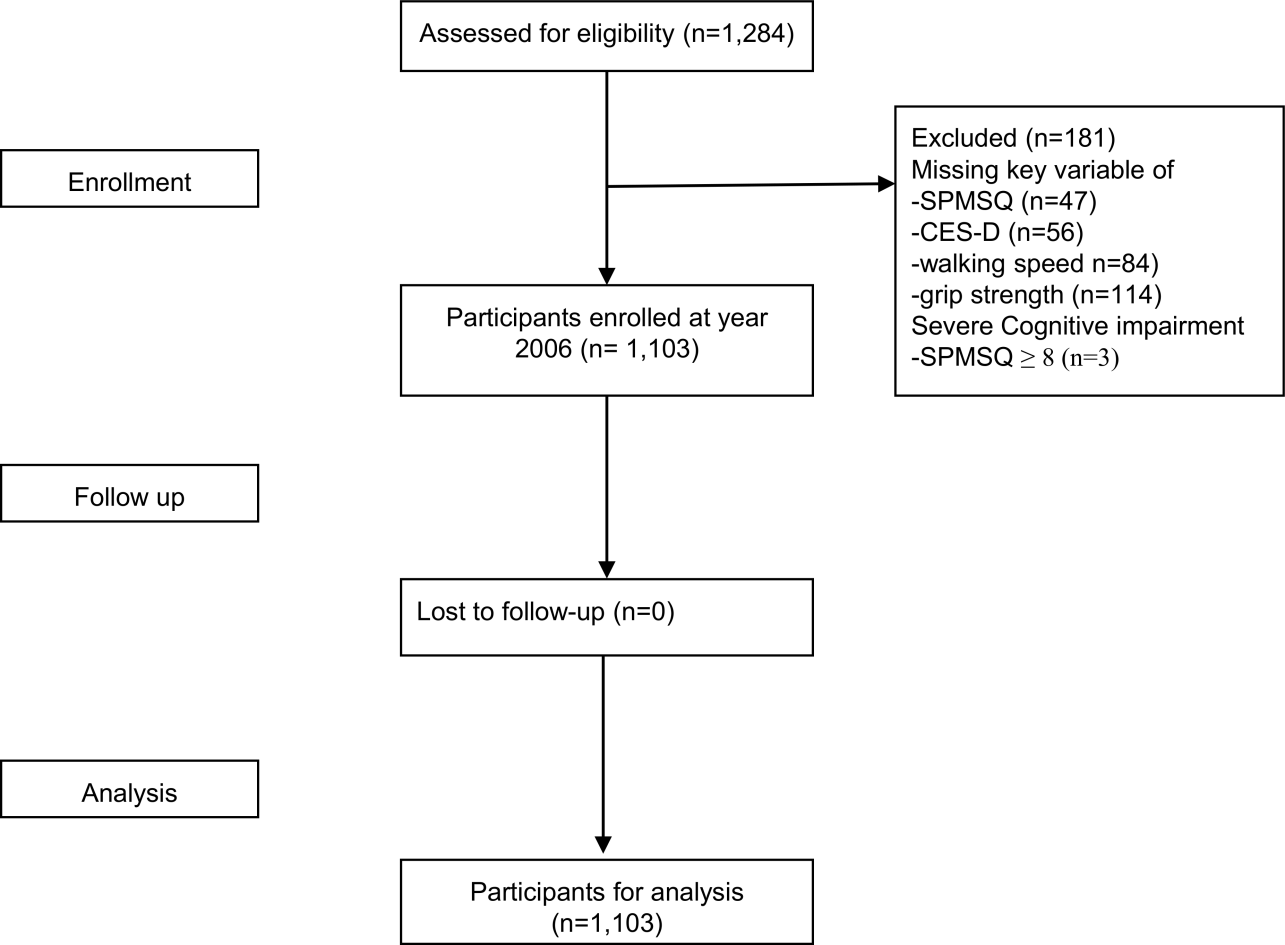
Participants derived from the Social Environment and Biomarkers of Aging Study 2006.

Data from the second wave of the Social Environment and Biomarkers of Aging Study (SEABAS) in 2006 were retrieved for this study. SEBAS intended to explore the interrelationship between biopsychosocial factors and aging, which used multi-stage proportional-to-size sampling strategies to ensure the national representativeness of enrollees. Details of the study design, and data collection procedures have been published previously [14]. Briefly, 1,284 subjects were enrolled for SEBAS 2006 from the original 1,659 participants of SEBAS 2000, and all participants received face-to-face interviews by well-trained nurses. Data of 181 participants with data incompleteness were excluded, which left data of 1,103 participants for analysis (Fig 1). Although the excluded subjects were significantly older (70.0 versus 65.1 years, *p*<0.001), having fewer educational years (4.7 versus 7.4 years, *p*<0.001), more multimorbidity (Charlson Comorbidity Index: 1.0 versus 0.7, *p* = 0.003), there were no sex-differences in their demographic characteristics.

The observational design and reporting format of this study followed STROBE guidelines [15]. A written informed consent was obtained from every participant. The Joint Institutional Review Board of Taiwan approved the study protocol. The design and procedures of the study were carried out in accordance with the principles of the Declaration of Helsinki.

### Dynapenia and cognitive impairment

The North Coast™ hydraulic hand-dynamometer (NC70142, California, US) was used to measure dominant handgrip strength. The maximal reading of three trials was recorded, and weakness was defined by the Asian Working Group on Sarcopenia (AWGS) <26 kg for men and <18 kg for women [16]. Walking speed was measured by a 3-meter walking test, and slowness was defined as walking speed less than 0.8 m/s according to AWGS criteria [16]. Dynapnea was defined as the presence of slowness and/or weakness [12].

The Short Portable Mental Status Questionnaire (SPMSQ), an easy-handled and validated tool, was used to evaluate the cognitive performance for participants [17,18]. Participants had severe cognitive impairment (SPMSQ≥8) were excluded for analysis. Cognitive impairment was defined as the SPMSQ score less than 1.5 standard deviation or more below age-, sex-, and education adjusted norms in the same population, i.e. SPMSQ >1.7, and CF was defined as concomitant presence of dynapenia and cognitive impairment.

### Outcomes and follow-up

The main outcomes of the study was mortality. The date of death was identified from the Taiwan national death registry between their original interview and December 31 of 2010.

### Measurements for other covariates

Demographic characteristics of all participants, including age, sex and education years were collected. Smoking status was defined as tobacco consumption in the past six months. Participants who did exercise twenty minutes for twice or more per week were defined as those who carried on regular exercise. Multimorbidity was measured by the Charlson Comorbidity Index (CCI) [19]. Physical function was evaluated by the Katz Index of independence in activities of daily living (ADL) and the Lawton Instrumental Activities of Daily Living (IADL) [20,21]. Depressive symptoms was evaluated by the short version of Center for Epidemiological Studies Depression (CES-D) [22].

### Statistical analysis

In this study, numerical variables were expressed as means ± standard deviation and categorical variables were expressed as proportions. One-way ANOVA test was used to compare numerical differences between various combinations of dynapenia and cognitive impairment, and Chi square or Fisher Exact test were used compare categorical variables when appropriate. Prevalence of cognitive frailty was stratified by ages and the Cochran-Armitage Trend Test was used to test the underlying trend. Schoenfeld residuals were used to test proportionality assumptions of Cox proportional hazard models. Age, sex and education attainment adjusted- and full adjusted- Cox proportional hazard model was used to explore the association between CF and mortality risk. Rothman synergetic index was used to examine the synergistic effect of dynapenia and cognitive impairment on mortality risk [23]. Sensitivity analysis was conducted by (1) excluding participants died in first year, who might commit serious illness and (2) excluding those with any disability of ADL.

All analyses were performed with the SAS statistical package, version 9.4 for windows (SAS Institute, Inc., Cary, NC, USA). A two-sided *P*-value <0.05 was considered statistically significant.

## Results

Overall, the mean age of all participants was 65.1±9.5 years (from 53 to 85 years), and the prevalence of CF was 8.6% in the entire cohort, 2.2% in people aged 53–64 years, 10.2% in people aged 65–74 years and 22.7% in people aged 75 years and over. The prevalence of CF increased across age groups (*p* for trend <0.001). Table 1 summarized the baseline characteristics of the entire study cohort and compared differences between various CF conditions. Participants with CF were older, more likely to be women, less commonly to have regular exercise, having fewer educational years, more depressive symptoms and greater multimorbidity. Among those with dynapenia,179 (35.6%) posed both slowness and weakness.

**Table 1:** Baseline characteristics of all participants and those stratified by dyanpenia and cognitive impairment conditions.

	Total	Robust	Dynapenia(+) CI(-)	Dynapenia(-) CI(+)	Cognitive frailty	p value
n	1103	572(51.9)	408(37.0)	28(2.5%)	95(8.6%)	
Age	65.1±9.5	61.1±7.5	68.5±9.5	65.0±8.1	74.7±8.1	<0.001
Women	510(46.2)	220(38.5)	210(51.5)	16(57.1)	64(67.4)	<0.001
Smoke	222(20.1)	126(22.0)	82(20.1)	4(14.3)	10(10.5)	0.062
Exercise	444(40.2)	257(44.9)	155(38.0)	11(39.3)	21(22.1)	<0.001
Education	7.4±4.9	9.0±4.5	6.5±4.4	5.3±5.2	2.8±4.0	<0.001
SPMSQ	0.5±1.0	0.2±0.4	0.3±0.5	2.4±0.9	2.9±1.3	<0.001
CES-D	4.5±5.4	3.2±4.2	5.3±5.8	5.1±5.9	8.4±7.0	<0.001
Charlson Cormobility Index	0.7±1.0	0.5±0.9	0.9±1.1	0.8±1.3	1.1±1.2	<0.001
Walking speed	0.9±0.3	1.1±0.2	0.7±0.2	1.0±0.1	0.5±0.2	<0.001
Grip strength	27.5±10.4	32.5±9.2	22.9±8.9	27.0±7.1	16.8±7.1	<0.001
ADL	5.9±0.6	6.0±0.1	5.9±0.6	5.7±1.2	5.5±1.3	<0.001
IADL	5.5±1.2	5.9±0.4	5.3±1.2	5.3±1.4	3.8±1.9	<0.001
any ADL disable	52(4.7)	3(0.5)	24(5.9)	3(10.7)	22(23.2)	<0.001
any IADL disable	304(27.6)	55(9.6)	167(40.9)	8(28.6)	74(77.9)	<0.001
Hypertension	362(32.8)	152(26.6)	164(40.2)	7(25.0)	39(41.1)	<0.001
Diabetes	173(15.7)	66(11.5)	71(17.4)	6(21.4)	30(31.6)	<0.001
Stroke	40(3.63)	7(1.2)	22(5.4)	2(7.1)	9(9.5)	<0.001
Heart disease	184(16.7)	55(9.6)	98(24.0)	4(14.3)	27(28.4)	<0.001
Kidney disease	54(4.9)	22(3.9)	23(5.6)	2(7.1)	7(7.4)	<0.001

Plus-minus values are means ± standard deviation; n(%) are numbers(percentage); CI denotes cognitive impairment; SPMSQ denotes Short Portable Mental State Questionnaire; CES-D denotes Center for Epidemiologic Studies Depression Scale; ADL denotes activity of daily living; IADL denotes instrumental activity of daily living.

### Survival analysis

**Fig 2:**
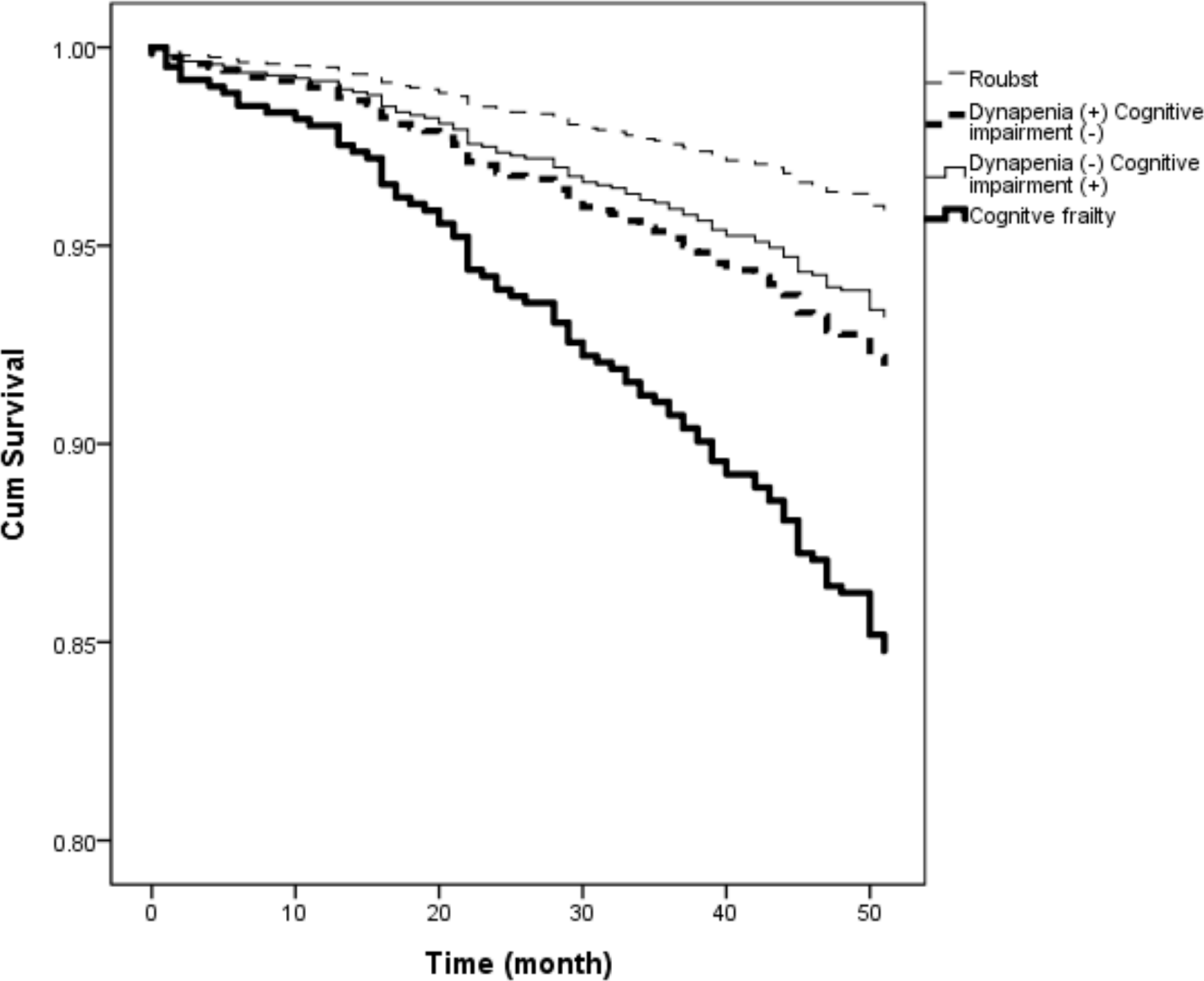
Age, sex and education adjusted survival plot for cognitive frailty.

During the median follow-up for 4 years, 88 participants died (2.0 per 100 person-years). Age, sex and education attainment adjusted survival plot showed that CF had highest risk for 4-year mortality. (Fig 2) Table 2 showed the association between CF and all-cause in the multivariate Cox proportional hazard model. Compared to robust individuals, hazard ratio (HR) of CF for all-cause mortality were 3.1 (95% CI:1.3–7.7). Rothman synergetic index for all-cause mortality were 2.8 (95% CI: 0.1–72.3), which showed synergistic effects of dynapenia and cognitive impairment on mortality risk.

**Table 2:** Cox proportional hazard model for all-cause deaths according to status of dynapnea and cognitive impairment.

	All causes		
		Model I	Model II
	Death/Total	HR (95%CI),p	HR (95%CI),p
	88/1103		
Robust	22/572	1	1
Dynapenia(+) CI(-)	42/408	1.8(0.9–3.3),0.078	1.6(0.8–3.1),0.145
Dynapenia(-) CI(+)	2/28	1.6(0.2–12.3),0.640	1.1(0.1–8.9),0.900
Cognitive frailty	22/95	4.1(1.8–9.2),<0.001	3.1(1.3–7.7),0.012
**Exclude participants die within first year**			
	76/1091		
Robust	20/570	1	1
Dynapenia(+) CI(-)	36/402	1.6(0.8–3.1),0.159	1.5(0.8–2.9),0.226
Dynapenia(-) CI(+)	1/27	0.0(0.0-.),0.988	0.0(0.0-.),0.985
Cognitive frailty	19/92	3.4(1.5–8.0),0.004	2.7(1.1–6.9),0.038
	74/1051		
Robust	20/569	1	1
Dynapenia(+) CI(-)	37/384	2.0(1.0–3.8),0.041	1.9(0.9–3.7),0.071
Dynapenia(-) CI(+)	2/25	2.1(0.3–16.4),0.462	2.2(0.3–16.7),0.452
Cognitive frailty	15/73	4.0(1.6–9.8),0.002	3.4(1.3–8.9),0.015

CI denotes cognitive impairment; HR denotes hazard ratios.

Model I adjusted for age, sex and educational years

Model II adjusted for Model I plus smoke, exercise, Center for Epidemiologic Studies Depression Scale, activity of daily living, instrumental activity of daily living, and Charlson comorbidity index

### Sensitive analysis

The association between CF and mortality risk remained robust when participants who died in the first follow-up year were excluded. The HR of CF for all-cause were 2.7 (95% CI:1.1–6.9). Further analysis for exclusion of baseline physical disability also confirmed the association between CF and all-cause death (HR: 3.4, 95% CI: 1.3–8.9).

## Discussion

In this study, the prevalence of CF was 8.6% based on the new diagnostic criteria and CF significantly predicted all-cause mortality. The association between CF and mortality risk remained strong after exclusion of baseline physical disability and those who died in the first-year follow-up period. Moreover, the dynapenia and cognitive impairment showed positive synergetic effect on all-cause mortality risk. The prevalence of CF was higher than that from previous studies using IANA/IAGG definition (ranging from 0.9% to 2.5%), but the demographic characteristics of CF subjects were in line with previous studies, i.e. older, less educated, and more often to be women [24–26]. Results of this study supported this new diagnostic criteria of CF by identifying reasonable numbers of people at risk for adverse health outcome, and may be early enough for disability and dementia prevention.

It has been reported that physical frailty and age-related cognitive impairment may share some common risk factors, such as depression and many cardiometabolic risk factors [27–29]. In this study, CF subjects had more depressive symptoms, higher prevalence of diabetes mellitus, hypertension, heart and kidney diseases, which was compatible with previous findings. In the Three-City Study, adding cognitive impairment to physical frailty improved the predictive validity for adverse health outcomes.[30] A study of 1,815 Mexican Americans showed that mortality risk of frail older people increased in the presence of cognitive impairment and vice versa [31]. Cumulative effects of cognitive impairment and frailty on mortality were reported in a Canadian cohort of 5-year follow-up and a Korean cohort of 3-year follow-up [32, 33].Results of this study extended previous findings to dynapenia with cognitive impairment in predicting all-cause mortality [26–34].

Ruan, et al., proposed to modify IANA/IAGG definition by using pre-frailty as a diagnostic component instead of physical frailty due to better intervention outcomes [10]. However, we proposed using dynapenia (slowness and/or weakness) instead of random combinations of other components of physical frailty to construct CF definition and aimed to explore the pathophysiology of CF [9, 11–13, 29]. It has been reported that weakness and slowness were the first emerging components of physical frailty [9], and results from the latent class analysis revealed that slowness and weakness was the commonest and strongest cluster of physical frailty, and was strongly associated adverse clinical outcomes [11]. This clustering phenomenon was also supported by the brain MRI imaging study [13]. For cognitive components of CF, researchers suggested using 1.5 standard deviation below the age- gender- and education- adjusted norms of cognitive tests instead of MCI [6,10,25]. Based on the definition, the prevalence of dynapenia without cognitive impairment was higher than the cognitive impairment without dynapenia in this study, which was in line with previous studies[35,36]. Therefore, the new operational definition of CF we used in this study was completely compatible with suggestions from previous studies, which also identified reasonable numbers of people and clearly demonstrated the association with adverse outcomes.

Despite all the efforts went into this study, there were still some limitations. First, data of other adverse health outcomes, e.g. new-onset disability, incident dementia, and healthcare utilization, were not available in this study, which may underestimate the diagnostic impact of CF. However, the survival status per se may sufficiently construct a conceptual model of CF. Second, no comprehensive neuropsychological and diagnostic evaluation of all neurodegenerative conditions were performed for study participants. However, participants with established diagnosis of neurodegenerative diseases would be excluded in the SEBAS recruitment process. Third, this study used only SPMSQ to identified subjects with cognitive impairment instead of measurements of individual cognitive domains. However, we believe this approach would be sufficient to identify cognitive impairment in various dimensions. Canevelli, et al., have indicated that there have been no robust/consistent operational definition of cognitive impairment in available studies [3]. Therefore, we believed we have done the best arrangement to identify early cognitive impairment for CF diagnosis.

## Conclusions

In conclusion, the prevalence of CF among community-living milled-aged and older people in Taiwan was 8.6% from a national representative cohort, and the individuals with CF were at significant risk for all-cause mortality. Further study is needed to evaluate the benefits of CF intervention programs and to explore the underlying pathophysiology of CF.
